# Equine exercise physiology—challenges to the respiratory system

**DOI:** 10.1093/af/vfac035

**Published:** 2022-06-14

**Authors:** Melissa Mazan

**Affiliations:** Clinical Sciences, Tufts University Cummings School of Veterinary Medicine, 200 Westborough Road, North Grafton, MA 01536, USA

**Keywords:** asthma, EIPH, equine athlete, exercise, respiratory

**Implications**

• Horses are remarkable athletes, whose respiratory systems are supremely capable of delivering high airflows and the oxygen that is necessary to superior athletic performance.

• Both nature and environment interfere with this oxygen delivery, resulting in poor performance, in the form of both upper and lower airway diseases.

• Horses have peculiar constraints to their breathing, such as being obligate nose breathers, that prevent them from ventilating fully during maximal exercise.

• Upper airway functional abnormalities such as laryngeal neuropathy, dorsal displacement of the soft palate, and nasopharyngeal collapse make this ventilatory challenge worse.

• Lower airway diseases, including equine asthma and exercise-induced pulmonary hemorrhage are pervasive in equine athletes, and further compromise athletic performance.

## Pulmonary Function During Exercise

The job of the respiratory system is, ultimately, to deliver oxygen to, and take carbon dioxide away from the rest of the body. It is important, therefore, to refresh our memories about the transport of oxygen in the body.

### Oxygen transport and oxygen-carrying ability of hemoglobin

**Figure 1.**
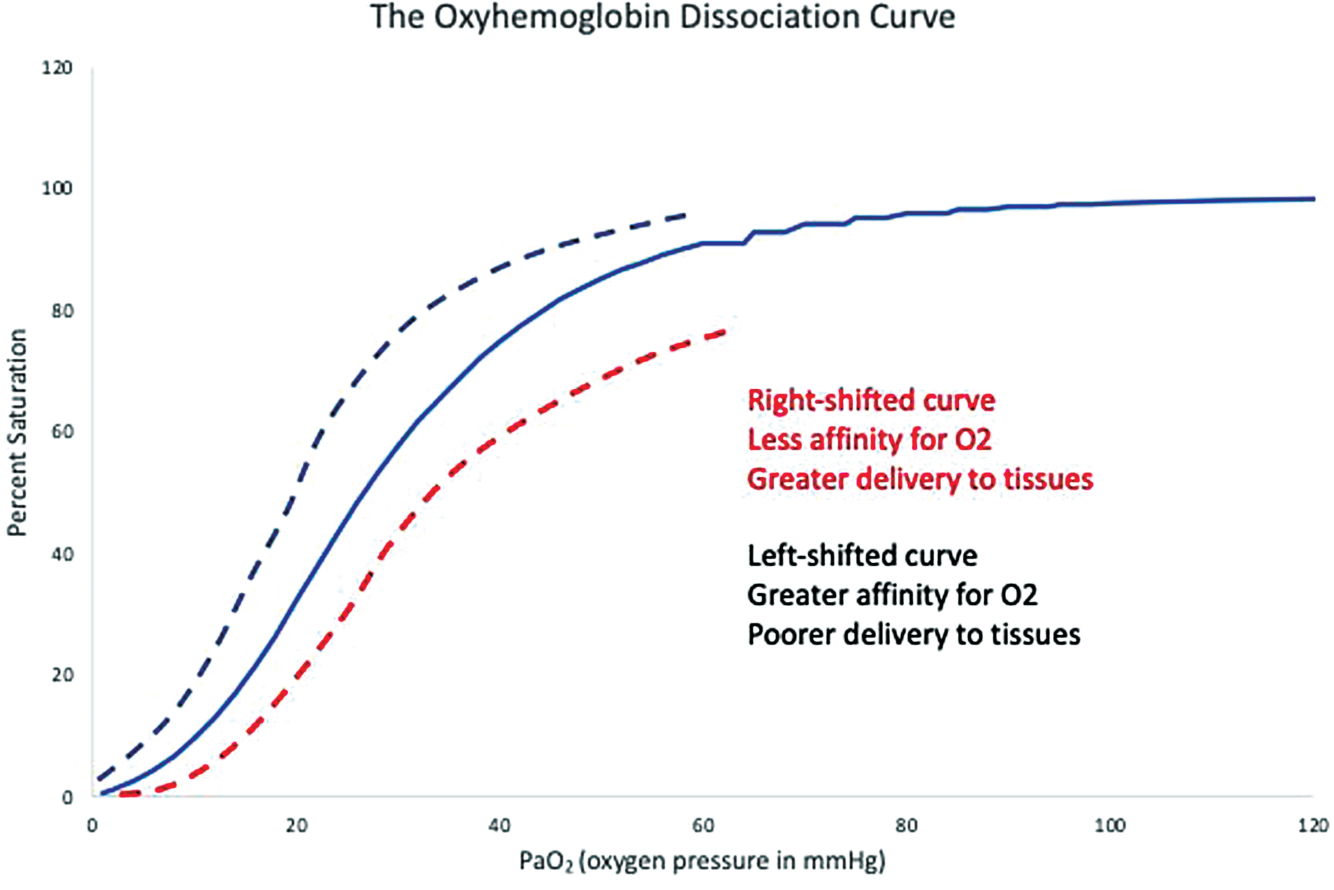
The oxygen dissociation curve shows the partial pressure of oxygen in the blood on the x-axis, and the percent saturation of hemoglobin on the y-axis. The sigmoid, or S-shaped, curve shows the effect of cooperative binding—hemoglobin has four subunits, and as each one binds a molecule of oxygen, the next subunit binds oxygen even more readily. Eventually, all the oxygen is saturated, so increases in oxygen pressure no longer cause any change. The curve can be shifted to the right, meaning that at any given PaO_2_, hemoglobin offloads oxygen more readily, making it more available to the tissues. The conditions that cause this are those found with both exercise and infection: increased temperature, increased PaCO_2_, and increased acidity. Conversely, a shift to the left is seen with decreased temperature, decreased PaCO_2_, and decreased acidity, and hemoglobin holds tight to the oxygen, making it less available to the tissues.

The question of how we get oxygen—21% of the air that we breathe every moment of our lives—to the cells of our body is one that deserves some consideration. Oxygen must travel in the blood, but very little oxygen can be transported as a dissolved gas in the blood—indeed, we would only be able to live for a few seconds on the amount of oxygen that is normally dissolved in the blood. Hemoglobin is an oxygen-carrying molecule that allows us to carry almost 70 times the amount of oxygen that is dissolved in the blood. Remember that each hemoglobin molecule has four iron molecules (Fe^2+^), each of which can carry one molecule of oxygen, and as the partial pressure of oxygen increases, hemoglobin becomes progressively more saturated until all four Fe^2+^ are carrying an oxygen molecule. It is important, also, to remember that the curve that describes the ability of hemoglobin to carry oxygen—that is, the oxygen hemoglobin dissociation curve (Figure 1)—is sigmoid, or S-shaped. In general, oxygen loading in the lungs takes place over the flat portion of the curve, whereas oxygen unloading in the tissues occurs over the steep portion of the curve (Marlin and Nankervis, 2002).

An important point to note about this curve is that the precipitously steep portion of the curve begins at an oxygen pressure—known as PaO_2_—of about 60 mmHg, whereas the normal individual of any species has a much higher resting PaO_2_ of around 98 to 100 mmHg. At this point, saturation of hemoglobin is still above 90%—but very small changes in PaO_2_ will cause a sharp decline in hemoglobin saturation.

### What affects this curve?

First, let’s think about what curve shifts mean. If the curve shifts to the right, then for a given O_2_ pressure, we have a lower affinity of hemoglobin for oxygen. This means that oxygen is *downloaded* more effectively, and is better able to participate in cellular metabolism. Things that cause the curve to shift to the right include:

- Increased acidity—and horses are remarkably able to function with high lactic acid and carbon dioxide levels in their bodies, which causes even more profound downloading of oxygen.
- Increased temperature—horses have a stunning increase in core body temperature, from about 100 °F at rest to about 107 °F at peak exercise, again, resulting in better downloading of oxygen.

In short, all mammalian species are better at downloading oxygen during exercise, but horses are particularly good (Fenger et al., 2000).

### What about myoglobin?

Myoglobin is a muscle protein with oxygen-carrying characteristics. Unlike hemoglobin, each myoglobin molecule has one site for reversible oxygen binding—this results in a hyperbolic curve, rather than a sigmoid curve. What does this mean? It means that myoglobin binds and retains oxygen at low pressures to a far greater degree than does hemoglobin. Thus, when oxygen levels are high, myoglobin hangs onto oxygen quite tightly. When oxygen levels drop, myoglobin is available as an extra source of oxygen. Although we seldom consider myoglobin as an important oxygen-carrying source in the resting individual, it becomes quite important during exercise. It is especially important for transporting oxygen to the mitochondria at the beginning of exercise and during very strenuous exercise (Garry et al., 2000).

### Ventilatory control during exercise

There is a large variety of inputs that determine the rate and depth of breathing necessary to the body’s needs at any particular level of rest or exercise. In most species, with the horse being a notable exception (and we will talk about this in due course), oxygen and carbon dioxide levels and blood acidity remain normal even during strenuous exercise. What allows this to happen? A combination of input from the respiratory center in the brain, the chemical state of the blood as it communicates with the brain, and the plasma oxygen, carbon dioxide, and acid levels.

### What effect does plasma PaO_2_ have on ventilation?

It is not surprising that if we breathe in a gas with a higher percentage of O_2_, our respiratory rate slows down, and if we breathe in a gas with a lower percentage of O_2_, our respiratory rate will increase. This is governed by chemoreceptors that are found at the arch of the aorta (*aortic bodies*) and at the branching of the carotid arteries (*carotid bodies*) (Marlin and Nankervis, 2002).

### What effect does plasma acidity have on ventilation?

The most important stimulus to ventilation is the partial pressure of carbon dioxide in plasma. Increases in CO_2_ directly stimulate chemoreceptors in the brain, areas that sense increases in acidity, and this stimulation causes the horse to increase the rate and depth of its breathing. Interestingly, however, ventilation increases almost immediately during exercise, beyond any increases in CO_2_ in the blood. It is thought that this may be from:

- brain input—that is, the excitement of beginning or impending exercise increases ventilation
- peripheral input from mechanical receptors in the joints.

During exercise, both the depth and rate of breathing are increased. Increased depth is important, as it delivers more oxygen to the body while getting rid of more CO_2_. Increased rate not only delivers greater minute volume, but likely helps to dissipate heat (Hodgson et al., 1994).

### How do we assess ventilation during exercise?

#### Steady-rate exercise.

During steady-rate, aerobic exercise, we find that ventilation increases as oxygen uptake does. It is often useful to look at the *ventilatory equivalent for oxygen* or the ${\dot{V}}_{E}/\dot{V}O_{2}$. This tells us about the efficiency of ventilation—essentially, how many liters of air must the individual breathe for every liter of oxygen that is consumed. We can also look at the ventilatory equivalent for CO_2_—during steady-state, aerobic exercise, this remains fairly constant, as the ventilation is more than adequate to rid the body of metabolically produced CO_2_ in most species—*except the horse* (Bayly et al., 1989).

#### Nonsteady-rate exercise.

If exercise intensity increases, there will eventually be a point at which tissue demand for oxygen has outstripped the combined respiratory and cardiovascular ability to supply oxygen. There will be a significant component of anaerobic metabolism, and lactate levels will begin to rise, along with increased tissue CO_2_ levels. This is termed the *lactate threshold*. (This is because sodium bicarbonate buffers the lactate that is produced during anaerobic metabolism, with subsequent production of water and CO_2_.) Ventilation must now increase to rid the body of CO_2_—both the demand to increase oxygen levels and decrease carbon dioxide levels will increase. This is termed the *ventilatory threshold* (Gondim et al., 2007).

### VO_2_max

**Figure 2.**
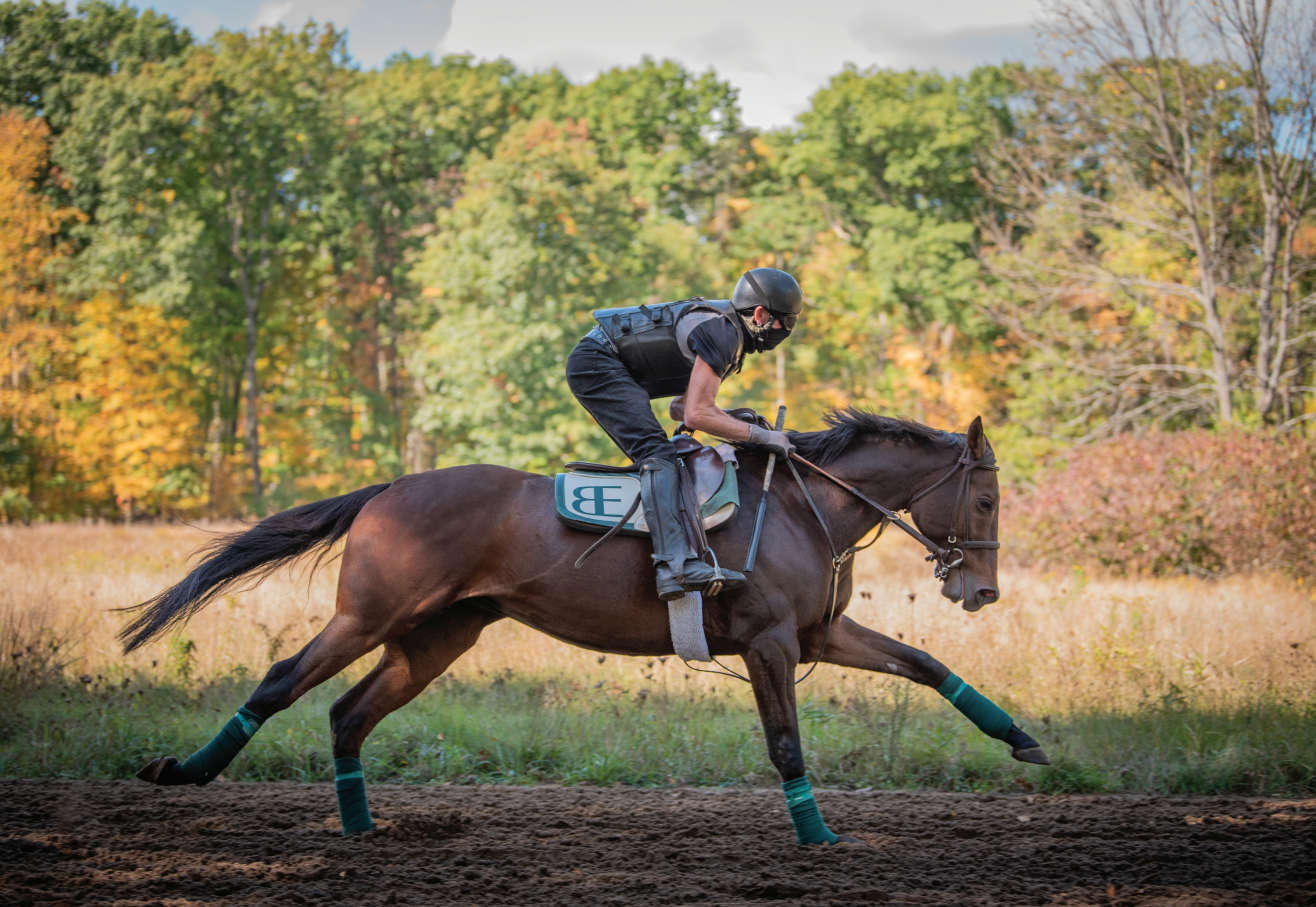
This Thoroughbred racehorse is breezing at the Burke Equine Training Track, Saratoga Springs, NY—and although he is traveling faster than any human will ever run, he is still far short of the 40 or so miles per hour that he can achieve in a race.

Horses are considered to be elite athletes—certainly, when we compare horses to people, we find that they are faster and have greater endurance. We can measure exercise capacity with respect to body weight to confirm that horses are not just faster because they are larger. Thoroughbreds, in particular, have impressive exercise capacity (Figure 2). One of the most useful ways of measuring an individual’s aerobic capacity—which is in turn a measure of athletic capacity—is to determine the body’s maximal capacity for oxygen consumption, or the **VO**_**2**_**max**. VO_2_ refers to the amount of oxygen that an individual takes up into the bloodstream at any given time. VO_2_ is really a flow rate, which is a *volume per unit of time.* In this case, we express the flow rate as *milliliters of oxygen per minute* (mL/min). We usually further determine the flow per unit of body weight (*milliliters of oxygen per kilogram per minute*, or mL/kg/min), so that we can *normalize* the measurement—that is, we can now compare large individuals to small individuals (Seeherman et al., 1981).

### What contributes to VO_2_max?

First, the airways are essential in transferring O_2_, to the lungs. Examples of airway problems that impede the delivery of oxygen to the lungs include *dynamic airway obstruction* and *equine asthma* (EA). Once the O_2_ is in the lungs, the blood vessels in the lungs must absorb the oxygen (and in return give up carbon dioxide, CO_2_), and bring this *oxygenated* blood to the heart. The heart must send the oxygenated blood to the rest of the body. A well-trained heart increases in size and weight, and, ultimately, in strength of contraction. During exercise, the muscles of motion receive the majority of available blood, whereas the blood supply to internal organs (such as the intestines and the kidney) is at a minimum. This requires a fine balance between dilation of blood vessels serving the muscles that are working, along with regulation of blood pressure to ensure delivery of blood where it is needed. Finally, the ultimate job of the blood supply is to deliver oxygen to mitochondria. It is actually the heart, capillarization of muscle, and density of mitochondria, and not the respiratory system, that contribute most to VO_2_max (Kearns et al., 2002).

While horses are inarguably elite athletes, they have some unusual constraints to their athletic performance. One of the most important of these is that despite their far higher VO_2_max than humans (180 mL/kg/min as opposed to 80 mL/kg/min in humans), their inability to switch to mouth-breathing to decrease resistance imposes a severe ventilatory limitation. In consequence, unlike all other species—except, perhaps, a few elite human athletes—horses develop hypoxemia (low blood oxygen) as well as hypercapnia (high CO_2_) during intense exercise, which will be more fully discussed below (Franklin et al., 2012).

#### Exercise and ventilation.

In horses, minute ventilation at rest is in the region of 80 L/min. During exercise, this may increase 10-fold, to a staggering 1,800 L/min. During the walk and trot, there does not appear to be a regular relationship between gait and respiration frequency. During the canter and gallop, however, breathing frequency and stride frequency are tightly coupled. This is also seen in humans who are trained distance runners (Fulton et al., 2018). This is probably due, in horses, to a combination of the abdomen and its contents acting like a piston, and the flexion of the back and thorax by the limbs. Thus, during the weight-bearing phase, the rib cage is compressed, facilitating exhalation. Although normal horses have intermittent noncoupled “big breaths” during exercise, lack of stride/breath coupling usually indicates respiratory disease (Attenburrow and Goss, 1994).

### Pulmonary blood flow during exercise

The phenomenon of *exercise-induced pulmonary hemorrhage* (EIPH) which is seen at least on a microscopic level in all horses that perform fast exercise, makes the study of pulmonary blood flow during exercise of more than passing interest in the horse. Interestingly, unlike the case in man, gravity seems to have little effect on distribution of blood flow in the horse. Rather, blood flow goes preferentially to the dorsal regions of the lung. This may be because of the way the pulmonary arterial tree branches and may also be due to the greater dilation of dorsal pulmonary arteries during exercise.

Horses have an unprecedented *pulmonary hypertension* during exercise, reaching close to 100 mmHg at the gallop (remember, resting pulmonary artery pressure is around 12 to 24 mmHg). It is not clear what causes this pulmonary hypertension, although it may be that the high left atrial pressures (necessary for filling the left ventricle at heart rates over 200 beats per minute) is an important contributor. Although there are many theories as to why horses develop EIPH, this pulmonary hypertension is probably the most important contributor (West et al., 1993; Sullivan et al., 2015).

### Exercise-induced hypoxemia

Studies have shown that even at a submaximal exercise intensity such as horses experience when working at 60% of a predetermined VO_2_max, arterial hypoxemia and hemoglobin desaturation develop. When we contemplate the causes (in general) of hypoxemia, we find that we have five basic mechanisms:

1. Decrease in inspired FiO_2_ (not a factor unless the horse is competing at a higher altitude)
2. Right to left shunts—meaning that blood that has not yet been oxygenated in the lungs is sent out to the body instead of oxygen-rich blood
3. V/Q mismatching—this refers to areas of the lung failing to match oxygen delivery with blood delivery
4. Diffusion impairment—this refers to the inability of oxygen to diffuse freely across thickened lung membranes
5. Alveolar hypoventilation—this refers to a failure of the individual to move sufficient air through the respiratory system, which can be seen when the horse does not breathe deeply enough, quickly enough, or a combination of the two

Because the difference between alveolar and arterial oxygen levels increases approximately 6- to 8-fold during exercise in horses, it is likely that diffusion impairment plays an important role in the very low oxygen levels that horses have at peak exercise (Marlin and Nankervis, 2002). This may, in turn, be due to a shortened transit time for oxygen through the pulmonary capillaries due to the impressively large increase (eight times normal) in cardiac output that the horse is capable of. Horses also likely hypoventilate—in support of this is the finding that when horses are given a less dense gas to breathe (for instance, helium–oxygen), the alveolar–arterial gradient decreases as well as the development of hypercapnia, or high carbon dioxide levels in the blood. This is truly of interest, as all other species that have been studied hyperventilate in order to normalize or even lower blood carbon dioxide (Wagner et al., 1989; Durand and Raberin, 2021).

### What about pulmonary mechanics in exercising horses?

The work of breathing (WOB) is negligible in the resting individual but becomes very important during exercise. It seems obvious that the respiratory system must work harder in expanding the lungs and chest wall, and in overcoming the resistance of the airways. What is not so intuitive is that the WOB also increases during exercise due to *inertance*—this describes the impedance, or opposition, to breathing that moving a column of air back and forth at increasing frequencies entails (Art and Lekeux, 1989).

### Frictional resistance

It is important not to lose sight of the fact that horses are ***obligate nose breathers***—they cannot switch to oronasal breathing, as can most other species. When we remember that the nasal passages contribute approximately 50% of the airways’ resistance, this becomes extremely important. One of the first adaptations that we humans make when we begin to exercise is to open our mouths. If you want to know what it feels like to be an obligate nose breather, try running in place with your mouth shut. The urge to open your mouth and thus decrease resistance is almost irresistible. Despite this limitation, the horse’s respiratory system makes many attempts to decrease resistance. The external nares dilate considerably, the larynx abducts and opens fully, and there is a degree of bronchodilation even in normal horses. However, the friction and turbulence engendered by large increases in air speed opposes these adaptations. The upshot is that, again, unlike other species, resistance actually *increases* during exercise in horses (Franklin et al., 2012).

**Figure 3.**
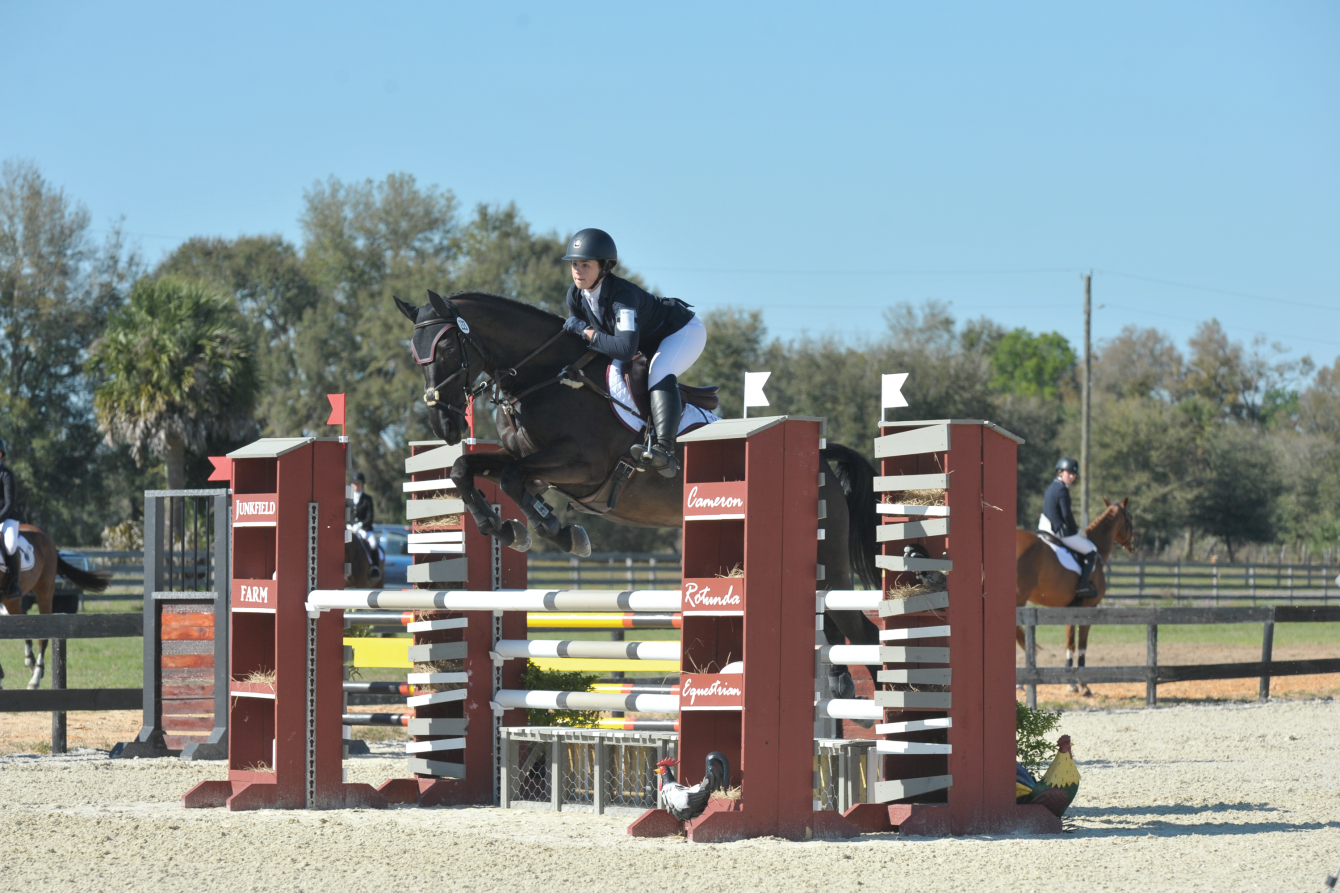
Horses naturally bend their necks and backs when they jump, as seen with this Warmblood. While this enhances their ability to jump, it increases respiratory resistance (Katerina Garcia-Chope and Samba Dromo).

Often unknowingly, we as riders add to frictional resistance. Horses naturally want to extend their heads and necks during exercise—and it makes sense that they should, because this maneuver stiffens the trachea, and prevents it from collapsing. (Surprisingly, horses generate such high flows, with resultant negative pressure, in their upper airways during exercise, that even supposedly rigid structures, such as the trachea, can collapse.) In disciplines such as dressage, jumping (Figure 3), and equitation, we ask the horses to put a large bend in the extra-thoracic airway at the level of the larynx and upper trachea—this causes a narrowing of the lumen, invoking Bernoulli’s principle, and an even more negative airway pressure, thus leading to dynamic collapse and eventually lower airflows. That said, there is some evidence that upper airway collapse happens even in nonridden horses, so the contributions of genetics, anatomy, and innervation cannot be ignored (Strand et al., 2012).

### So what does all this mean for the horse?

When the horse is galloping, WOB increases markedly. Indeed, the WOB per minute increases almost 500-fold between rest and exercise. Although most texts on exercise physiology based on humans hold to the basic tenet that ventilation is not a limiting factor in exercise in the normal individual, the case is probably different in the horse. This type of impressive increase in WOB with exercise strongly suggests that the horse may reach a point where increases in ventilation cost more in energy than the horse can afford while still performing mechanical work (Art and Lekeux, 1989).

## Some Specific Respiratory Diseases of the Horse That Affect Exercise

### Upper respiratory diseases

Horses, as alluded to above, have the peculiar anatomical makeup that demands that the animal breathe through the nose, and not the mouth. Thus, the horse is an *obligate nose breather.* Most ruminants are preferential nose breathers—cattle, sheep, and goats, for instance, prefer to breathe through their noses, but can breathe through their mouths in case of necessity. Human infants are not obligate nose breathers—but they prefer to breathe through their noses and must detach the soft palate from the tongue in order to breathe through their mouths. It is only with maturity and descent of the larynx that we humans begin to find it easier to breathe through our mouths during exercise—and any parent who has had a baby with a respiratory virus and has syringed the mucus out of a baby’s nose can attest to this anatomical demand. For horses, the requirement to breathe through the nose is strict (Rodenstein et al., 1985).

Moreover, horses, during exercise, must move tremendous amounts of air—upwards of 1,800 L/min—several bathtubs worth—through the nostrils, which imposes a significant resistive load on the respiratory system. As this large flux of air travels through the rigid structure of the nose, larynx, and trachea, very large negative pressures are generated, which only the most perfect of upper airways can withstand. Any weakness within the system results in the collapse demanded by Bernoulli’s theorem—which states that as speed of a fluid—or gas—increases, then the pressure must decrease.

**Figure 4.**
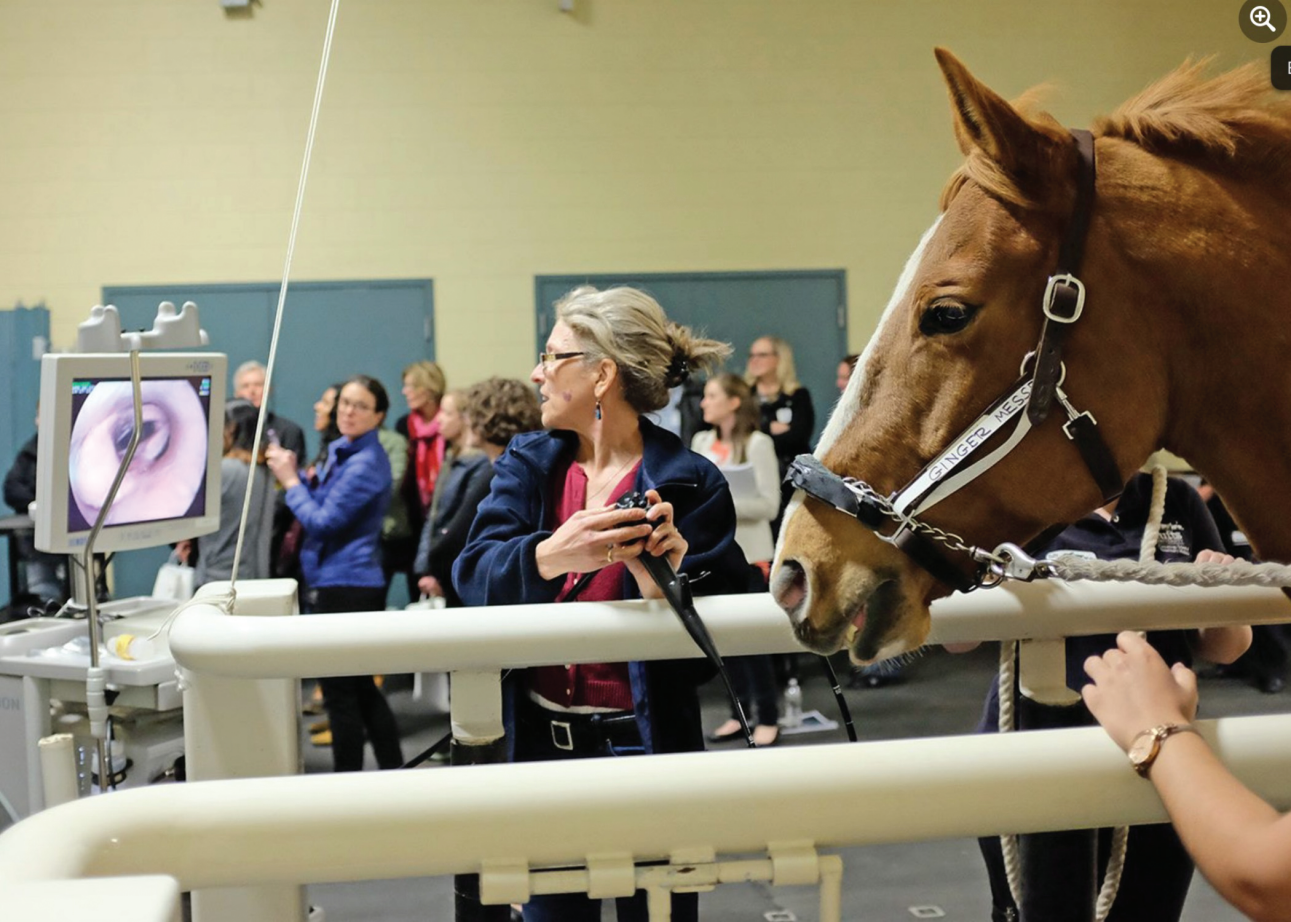
The author performing dynamic endoscopy on the treadmill at Tufts Cummings School of Veterinary Medicine Hospital for Large Animals. This horse was referred for possible laryngeal hemiplegia, but had a normal airway at exercise—she ended up having equine asthma, which was treated successfully.

The upper airways of the horse are meant to be rigid, but there are a few important abnormalities of the upper airway that result in floppiness of the upper airways, with resultant dynamic collapse that results in high resistances, high WOB, and poor performance. The most common of these are laryngeal paralysis, dorsal displacement of the soft palate (DDSP), and nasopharyngeal collapse; epiglottic entrapment is also seen, but is much less common. These conditions are often only seen during exercise, necessitating dynamic upper airway endoscopy (Figure 4).

The term *laryngeal neuropathy*, refers to the curiosity that the left side, and only the left side, of the larynx is neurologically dysfunctional in over 95% of horses. It is supposed that this is because the nerve on the left side is considerably longer and has a more circuitous route than that on the right side. The part of the larynx that is affected is the arytenoid cartilage, which should serve to open the larynx during inspiration, especially during exercise, but fails to do so in horses with laryngeal neuropathy. There is a wide spectrum of disease, with those who are mildly affected often having a discernable delay in opening of the airway at rest, but full function at exercise, whereas those that are severely affected are not able to overcome the deficit. These horses make a characteristic “roaring” noise which, on its own, is of no concern, but the resistance presented by the paralyzed left side of the larynx and the ensuing increase in WOB causes decreased performance in horses going at speed. Surgical approaches to the problem exist; unless the horse is truly working at speed, the problem is more of a nuisance or aesthetic than anything else (Parente, 2018).

DDSP is peculiar to the horse. Horses, as obligate nose breathers, have a configuration of the upper airways that places the larynx dorsal to, or on top of, the soft palate. This is distinctly different to humans and most other mammals, in which the soft palate lies below the larynx. The cause of DDSP is not fully understood, although it is apparent that in most horses with DDSP there is abnormal contraction of the muscles governing the soft palate. The end result is, again, an abnormal noise, increased respiratory resistance, and increased WOB leading to poor performance. Surgical approaches are commonly employed (Cercone et al., 2019).

A less common upper airway abnormality is epiglottic entrapment—surely an oddity. In this upper airway disease, the epiglottis, the triangular piece of cartilage at the base of the larynx, becomes entrapped in the surrounding tissue and—not surprisingly—the laryngeal aperture is narrowed, resistance increases, WOB increases, and the horse fails to perform as expected. Surgery is remarkably effective for this affliction, with few relapses (Saulez and Gummow, 2009).

### Lower airway disease

The lower airways can be considered the final gatekeeper before oxygen enters the blood and carbon dioxide exits. Horses are blessed with large airways and lungs that allow them to be superb athletes, but the downside of this largesse on the part of evolution is that unless they are truly elite athletes, they may withstand noninfectious disease of the lower respiratory tract for months to years before the owner or trainer notices. The two conditions of the lower respiratory tract that affect the athletic horse during exercise are EIPH and EA (formerly known as inflammatory airway disease). The former may be considered, at least at the onset, as a problem of physiology rather than a disease, and the latter is a disease primarily of domestication: both are widespread among the athletic horse population and account for an impressive number of horses that fail to perform to their potential. Because of the high demands for oxygen in the athletic horse, even minor insults to the oxygen-carrying capacity of the body can affect performance, so it is of critical importance to keep the lungs as healthy as possible.

#### Exercise-induced pulmonary hemorrhage.

EIPH, or bleeding that comes from the horse’s lungs during exercise, is certainly the most dramatic of the lower airway diseases that affect equine athletes, and the one that causes most consternation in the lay population. It is commonly held both by veterinarians and the lay population that EIPH is a major cause of wastage for equine athletes, especially in the racing industry, and that, when severe, it can result in sudden death. Despite this accepted knowledge, in reality, the connection between performance and EIPH is still poorly understood and sudden death due to EIPH occurs in less than 0.029% of the racing population. There is, indeed, debate as to whether EIPH is even a disease, or whether it is an inevitable outcome of the remarkable physiology that allows the horse to be an elite athlete (Crispe and Lester, 2019).

#### Epidemiology—who gets it and what are the risk factors?

Bleeding Childers, the grand-progenitor of Eclipse, who is ubiquitous in the pedigree of almost all modern racehorses, was eponymously named because he bled so often (Poole and Erickson, 2016). Although EIPH is well-recognized in the equine racing population, with the vast majority of racing Thoroughbreds and Standardbreds affected, it has become increasingly clear that this is a disease of any horse that performs strenuous exercise at speed. Interestingly, populations of horses that perform bursts of work at speed are those that have most recently been identified as experiencing EIPH, including barrel horses, whose runs generally last less than 20 s and polo ponies that run for short bursts of speed during a 7-min chukka. Reported prevalence is dependent on the method used for diagnosis (da Silva et al., 2017; Gold et al., 2018).

The biggest risk factor for a horse developing EIPH is going at high speed or undergoing very intense exercise or acceleration to full speed. This is most easily appreciated in Thoroughbred racehorses, where it has been found that more horses have evidence of pulmonary hemorrhage after racing than they do after breezing. Interestingly, horses jumping at speed, such as steeplechasers, are more likely to develop EIPH than horses that race on the flat. Although older horses are commonly thought to be more susceptible to EIPH, it is years spent racing rather than age itself that is associated with EIPH (Hinchcliff et al., 2015).

#### Pathogenesis—why do horses bleed from the lungs?

The prevailing theory for the cause of EIPH posits that stress failure of pulmonary capillaries occurs due to very high transmural pressures, or the pressure that develops across the wall of the pulmonary capillary. It is easiest to think of this as a push and a pull—the push comes from a very high pulmonary artery pressure at intense exercise, and the pull comes from a very negative alveolar pressure at the same time as the horse breathes in. This, in turn, is mandated in order to accommodate the horse’s phenomenal exercise capacity—in order to supply the amount of oxygen that the horse needs to perform at its extraordinary levels of VO_2_max, the heart rate must be high and pulmonary capillary pressures skyrocket from approximately 25 mmHg to up to 90 mmHg because high left ventricular filling pressures are necessary to maintain cardiac output in these conditions. Simultaneously, in order to accommodate the need for increased ventilation that is coupled to the horse’s stride, the respiratory system is forced to exert tremendous negative pressures in the pleural space—up to −60 cm H_2_O, or approximately 45 mmHg. These pressures summate to over 120 mmHg transmural pressure, making it easy to understand why pulmonary capillaries then rupture.

For perspective, in human athletes, 20 to 25 mmHg threshold for pulmonary capillary pressures is associated with interstitial lung edema and altered ventilation/perfusion relationships and maximum pulmonary arterial pressures of 40 to 50 mmHg, which elite human athletes can achieve at maximal exercise, are considered to correspond to the extreme of tolerable right ventricular afterload. These numbers seem positively puny with respect to the exercise physiology of the horse (West et al., 1993).

Anything, therefore, that increases either pulmonary artery pressures or decreases (creates a larger negative pressure) alveolar pressures will potentiate EIPH in the equine athlete. The most logical comorbidities that would contribute, therefore, would be dynamic upper airway obstructions, such as laryngeal hemiplegia or DDSP. The tendency of steeplechasers to hold their breath over the jump, thus causing a greater negative pressure during great exertion, may help to explain their increased likelihood to bleed. Human athletes that hold their breath, such as sprint swimmers and divers, also have an increased likelihood of experiencing alveolar hemorrhage (McKenzie, 2012).

#### EIPH—summary.

EIPH, at least in its early stages, is likely less a disease and more a consequence of the physiology that is mandated by the development of the horse as a superb athlete. The combination of the need for high left ventricular filling pressures in order to fulfill the demand for oxygen culminating in high pulmonary capillary pressures along with very large pleural and thus alveolar pressure swings results in mechanical stress and rupture of pulmonary capillaries. This explains the old adage that “if a horse doesn’t bleed it isn’t running fast enough.” Indeed, alveolar hemorrhage with exercise is not limited to horses—it seems to be the province of elite athletes, including greyhounds, racing camels, and some elite human athletes (Akbar et al., 1994; Epp et al., 2008).

### Equine asthma

Although EIPH is the most publicly recognized lower airway disease of sport horses, EA has a more pervasive and broad impact on the sport horse population. EA, as a disease of domestication and exposure to particulate matter, affects all groups of horses, including those, like endurance horses, that do not perform under conditions that produce high pulmonary artery pressures. EA is described as an inflammatory but *non*septic disease of the equine respiratory system, meaning that it is not due to viral or bacterial infection. The more severe form, recurrent airway obstruction, otherwise known as recurrent airway obstruction or heaves, will not be discussed here.

#### Epidemiology and etiology—who gets EA and what causes it?

Many different causes likely contribute to the constellation of signs that we recognize as EA. The most commonly invoked contributors remain high levels of particulates in the environment, viral disease, air pollution, genetic predisposition, and bacterial infection.

It has long been noted that racehorses with clinical signs of EA often live in poorly ventilated stables, and organic dusts and molds are at least partly to blame. Organic dust in the barn environment, including hay, manure, shavings, molds, fungi, animal dander, mites, and plant material, is very good at eliciting an inflammatory response, and ammonia, which can be extremely high even in well-managed stables, contributes to lung inflammation and damage. Endotoxin and particulates likely act in concert to exert the most profound inflammatory effect. It is unsurprising that hay-eating has been identified as a risk factor for increased tracheal mucus in pleasure horses. The worst offender in increasing exposure to airborne particulates is the hay net, as it is directly in the horse’s breathing zone. Inorganic particulates are of less importance, but still contribute, with silicates from dusty arenas or oil fly ash from diesel machinery being used inside large barns (Couetil et al., 2020).

Veterinarians and trainers have long suspected that respiratory viral disease can trigger EA. Recent infection with a respiratory virus is the most common trigger for exacerbation of the similar disease, asthma, in humans, so it is logical to make the connection to EA, and evidence is building to support that hypothesis. A recent study in the author’s laboratory has shown that horses with hyperresponsive airways have a correspondingly higher titer to equine rhinitis A virus and to equine alphaherpesviruses. As immune responses to respiratory viruses are also frequently found in healthy horses, it is clear that a full understanding of the link between past infection and current disease is still to be determined (Houtsma et al., 2015).

The extent to which EA affects performance depends on the use of the horse and the expectations of the owner or trainer. Practitioners who work primarily with pleasure or show horses might report a low incidence of EA in young horses but will see more cases as horses age and the disease progresses. This is because these horses do not work sufficiently close to their VO_2_max to unmask subclinical EA. Practitioners working with young horses working near or at VO_2_max, such as racehorses, will notice exercise intolerance far more frequently. Cough, rather than overt exercise intolerance, is more commonly reported in sport horses other than racehorses (Bedenice et al., 2008).

EA is diagnosed through a combination of clinical signs, cytological examination of respiratory secretions, lung function, and response to treatment. The most important treatment for long-term success is environmental remediation. The barn environment is replete with organic particulate matter, respirable endotoxin, molds, and volatile gases such as ammonia. The worst offenders appear to be hay and straw. Multiple studies have shown that significant improvements can be made by replacing dusty substrates and feed with less dusty substitutes. For instance, pelleted hay and wood shavings are often better than regular hay and straw bedding, but outdoor living, in most cases, is the best. What is not clear to many owners is that even small or transient contacts with hay can initiate severe signs and should be avoided. In addition to changing to low dust feeds and beddings, the following recommendations to owners should be made (Couetil et al., 2020):

- Feed hay from the ground, not from a hay net
- Soak hay well before feeding or use ensiled or baked hay products
- Wet any dusty grain (e.g., pellets) before feeding
- Sprinkle aisle ways with water before sweeping
- Avoid storing hay overhead. If unavoidable, lay a tarp under the hay to avoid dust raining down on the horses
- Use a humectant or hygroscopic agent to reduce dust in the indoor and outdoor arenas
- Remove horses from the barn while cleaning stalls or moving hay
- Do not use blowers to clean aisles
- Remove cobwebs and other dust collectors routinely when horses are out of the barn

Despite our best efforts, it is almost impossible to achieve a respiratory-friendly barn environment, and horses with EA must be treated with corticosteroids and bronchodilators as well, similar to human athletes. The treatment of EA is beyond the scope of this article.

In summary, horses are remarkable athletes, whose respiratory systems are supremely capable of delivering high airflows and the oxygen that is necessary to superior athletic performance. Both nature and environment interfere with this oxygen delivery, resulting in poor performance, in the form of both upper and lower airway diseases.

## About the Author

**Dr Melissa Mazan** is a Professor of Large Animal Internal Medicine at Tufts Cummings School of Veterinary Medicine. As Director of the Tufts Bronchoalveolar Lavage Cytology Laboratory, Dr Mazan runs a busy nationwide BAL cytology service, and as Co-Director of the Tufts Equine Respiratory Health Laboratory, she provides one of the few comprehensive lung function testing services in the country. She did her undergraduate degree at Yale University, and completed her DVM at Tufts. She is a Diplomate of the American College of Veterinary Internal Medicine. Her research interests include development of new modalities for lung function testing and treatment of equine asthma, analysis of bronchoalveolar lavage cytology, and the equine respiratory microbiome, as well as the use of telemedicine in supporting the practice and teaching of equine medicine in developing countries.
